# Reduced thoracic skeletal muscle size is associated with adverse outcomes in diabetes patients with heart failure and reduced ejection fraction: quantitative analysis of sarcopenia by using cardiac MRI

**DOI:** 10.1186/s12933-023-02109-7

**Published:** 2024-01-13

**Authors:** Ke Shi, Ge Zhang, Hang Fu, Xue-Ming Li, Shi-Qin Yu, Rui Shi, Wei-Feng Yan, Wen-Lei Qian, Hua-Yan Xu, Yuan Li, Ying-Kun Guo, Zhi-Gang Yang

**Affiliations:** 1 Department of Radiology, Functional and Molecular Imaging Key Laboratory of Sichuan Province, West China Hospital, Sichuan University, Chengdu, Sichuan China; 2 Department of Radiology, Sichuan Cancer Center, School of Medicine, Sichuan Cancer Hospital and Institute, University of Electronic Science and Technology of China, Chengdu, Sichuan China; 3 Department of Radiology, Key Laboratory of Birth Defects and Related Diseases of Women and Children of Ministry of Education, West China Second University Hospital, Sichuan University, Chengdu, Sichuan China; 4 Laboratory of Cardiovascular Diseases, Regenerative Medicine Research Center, West China Hospital, Sichuan University, Chengdu, Sichuan China

**Keywords:** Diabetes mellitus, Heart failure with reduced ejection fraction, Sarcopenia, Cardiac MRI

## Abstract

**Background:**

Sarcopenia is frequently found in patients with heart failure with reduced ejection fraction (HFrEF) and is associated with reduced exercise capacity, poor quality of life and adverse outcomes. Recent evidence suggests that axial thoracic skeletal muscle size could be used as a surrogate to assess sarcopenia in HFrEF. Since diabetes mellitus (DM) is one of the most common comorbidities with HFrEF, we aimed to explore the potential association of axial thoracic skeletal muscle size with left ventricular (LV) remodeling and determine its prognostic significance in this condition.

**Methods:**

A total of 243 diabetes patients with HFrEF were included in this study. Bilateral axial thoracic skeletal muscle size was obtained using cardiac MRI. Patients were stratified by the tertiles of axial thoracic skeletal muscle index (SMI). LV structural and functional indices, as well as amino-terminal pro-B-type natriuretic peptide (NT-proBNP), were measured. The determinants of elevated NT-proBNP were assessed using linear regression analysis. The associations between thoracic SMI and clinical outcomes were assessed using a multivariable Cox proportional hazards model.

**Results:**

Patients in the lowest tertile of thoracic SMI displayed a deterioration in LV systolic strain in three components, together with an increase in LV mass and a heavier burden of myocardial fibrosis (all P < 0.05). Moreover, thoracic SMI (β = -0.25; P < 0.001), rather than body mass index (β = -0.04; P = 0.55), was independently associated with the level of NT-proBNP. The median follow-up duration was 33.6 months (IQR, 20.4–52.8 months). Patients with adverse outcomes showed a lower thoracic SMI (40.1 [34.3, 47.9] cm^2^/m^2^ vs. 45.3 [37.3, 55.0] cm^2^/m^2^; P < 0.05) but a similar BMI (P = 0.76) compared with those without adverse outcomes. A higher thoracic SMI indicated a lower risk of adverse outcomes (hazard ratio: 0.96; 95% confidence interval: 0.92–0.99; P = 0.01).

**Conclusions:**

With respect to diabetes patients with HFrEF, thoracic SMI is a novel alternative for evaluating muscle wasting in sarcopenia that can be obtained by a readily available routine cardiac MRI protocol. A reduction in thoracic skeletal muscle size predicts poor outcomes in the context of DM with HFrEF.

## Introduction

Sarcopenia, which was coined in 1989, is defined as the loss of skeletal muscle mass and function [1]. Patients with sarcopenia present with reduced exercise capacity and nutrition deficiency, leading to physical disability and deconditioning and subsequently adverse outcomes. The prevalence of sarcopenia in end-stage heart failure (HF) has been shown to be 20% higher than that in healthy subjects of the same age [2]. Actually, there may exist a deleterious interaction between sarcopenia and HF, with the reduced peripheral perfusion in HF triggering skeletal myopathy, while skeletal muscle degradation in turn deteriorates exercise capacity and thereby results in HF-related symptoms [3]. Recently, clinical evidence suggests that low axial thoracic skeletal muscle size is associated with the severity of HF and could predict adverse outcomes preceding overt body weight loss [4, 5]. Given this close relationship, sarcopenia is increasingly becoming recognized as an important abnormal body composition alteration, and evaluation of axial thoracic skeletal muscle size should be incorporated into the routine care of patients with HF [6].

Diabetes mellitus (DM) is one of the most common comorbidities in heart failure with reduced ejection fraction (HFrEF), which is present in up to 40% of these patients and portends a worse prognosis [7, 8]. It is necessary to better understand the potential association of sarcopenia with left ventricular (LV) remodeling and clinical outcome in patients with HFrEF and DM. However, to our knowledge, data on this issue are limited. Therefore, in this study, we aimed to (1) compare the clinical characteristics and MRI findings according to different grades of axial thoracic skeletal muscle size; (2) investigate the relationship between skeletal muscle size, LV remodeling, and plasma concentrations of natriuretic peptides; and (3) determine the prognostic significance of skeletal muscle size in diabetes patients with HFrEF.

## Methods

### Study population

The diagnosis of HFrEF was made according to the guidelines from the European Society of Cardiology (2021) [9]. Patients were initially enrolled between June 2015 and June 2022 with the presence of symptoms and/or signs of HF, an elevated amino-terminal pro-B-type natriuretic peptide (NT-proBNP), and a reduced LV ejection fraction (LVEF ≤ 40%). Patients were excluded whenever one of the following criteria was met: (1) age younger than 18 years old; (2) a history of decompensated HF within 3 months of enrollment; (3) acute coronary syndrome; (4) severe arrhythmia; or (5) incomplete clinical or MRI information. Demographics, clinical characteristics, and laboratory measurements at baseline were collected from electronic clinical records. DM status was then defined based on the current guidelines from the European Society of Cardiology (2019) [10]. This study was approved by the Biomedical Research Ethics Committees of West China Hospital and complied with the Declaration of Helsinki. Written informed consent was waived because of the retrospective nature of the study. All medical data were protected with full confidentiality and used only for the purpose of the present study.

### Cardiac MRI protocol

Cardiac MRI was performed on a 3-Tesla scanner (MAGNETOM Skyra/Tim Trio; Siemens Healthcare, Erlangen, Germany) for each patient at the time of HF diagnosis for evaluating cardiac structure and function. As included in the routine cardiac MRI protocol, an axial stack of steady-state free precession (SSFP) imaging was obtained with the following parameters: temporal resolution = 224.16 ms; echo time (TE) = 1.23 ms; slice thickness = 6.0 mm; flip angle (FA) = 60°; acquisition matrix = 126 × 256 pixels; and field of view (FOV) = 340 × 255 mm^2^. For cine imaging, a balanced SSFP sequence was performed with the following parameters: repetition time (TR) = 2.81 ms; TE = 1.22 ms; slice thickness = 8.0 mm; FA = 40°/50°; acquisition matrix = 166 × 208 pixels; and FOV = 340 × 284 mm^2^. Twenty-five frames were reconstructed per breath-hold acquisition for cine images. Fifteen minutes after the administration of gadolinium-based contrast (0.2 mL/kg), late gadolinium enhancement (LGE) imaging was acquired by phase-sensitive inversion recovery sequence. The acquisition parameters were as follows: TR = 700/500 ms; TE = 1.18/1.07 ms; slice thickness = 8.0 mm; FA = 40°; acquisition matrix = 184 × 256 pixels; and FOV = 350 × 295 mm^2^.

### Cardiac MRI imaging assessment

**Fig. 1:**
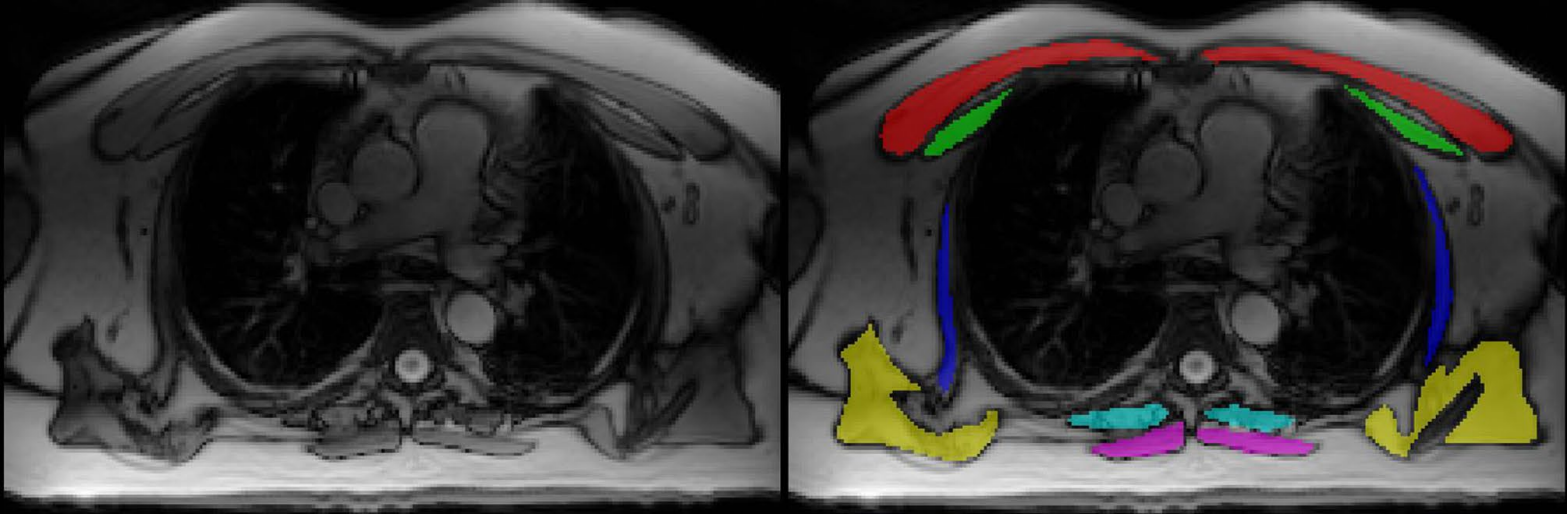
Axial MRI image at the level of carina demonstrating the measurements of bilateral cross-sectional area of thoracic skeletal muscle

All images were analyzed using commercially available CVI^42^ software (Circle Cardiovascular Imaging, Inc., Calgary, Alberta, Canada). Methods of axial thoracic skeletal muscle size measurements were based on the work conducted in a previous study [4]. In brief, thoracic skeletal muscle at the level of the carina, including muscle groups of pectoralis major, pectoralis minor, serratus anterior, periscapular, paraspinal, and trapezius muscles, were manually traced bilaterally to obtain cross-sectional area (i.e., skeletal muscle size [cm^2^]) (Fig. 1). The carina represents the inferior termination of the trachea into the right and left main bronchi. Periscapular muscles refer to the muscle groups of latissimus dorsi, subscapularis, and infraspinatus around the scapula. Axial thoracic skeletal muscle size in each patient was standardized with adjustment of body size and further produced skeletal muscle index (SMI [cm^2^/m^2^]).

For volumetric analyses, endocardial and epicardial borders were traced semiautomatically and manually corrected if needed at the LV end-diastolic and end-systolic phases in a series of short-axis images. LV function parameters, including EF, end-diastolic volume (EDV), end-systolic volume (ESV), and stroke volume (SV), were automatically calculated. LV papillary muscles were included in the LV mass (LVM) but not in the LV volume. For myocardial strain analyses, a stack of short-axis cine images combined with 4-, 2- and 3-chamber long-axis images were loaded into the feature-tracking module. We delineated LV endocardial and epicardial borders at the end-diastolic phases (reference phase) of all cine images. The software automatically traced the contours throughout the cardiac cycle. During the systolic phase, the LV shortens in the longitudinal and circumferential directions, causing negative global longitudinal peak strain (PS) and circumferential PS, whereas thickening in the radial direction causes positive global radial PS. Detection and quantification of LGE were carried out by both visual and quantitative methods using the established grayscale threshold method of six standard deviations exceeding the mean signal intensity of remote nonfibrotic myocardium.

To determine the interobserver reproducibility, half of the sample size selected in a random manner was analyzed by two experienced radiologists who were blind to each other’s findings. Moreover, to determine the intraobserver reproducibility, the same study sample was reanalyzed a second time 2 weeks after the initial assessment. Reproducibility was assessed with intraclass correlation coefficients (ICCs), which ranged from 0.88 to 0.92. ICCs > 0.80 indicated good reproducibility.

### Follow-up and outcomes

The primary composite outcome was recorded as HF hospitalization, cardiovascular mortality and heart transplantation, whichever occurred first. HF hospitalization was defined as an unplanned hospitalization or an urgent hospital visit for worsening HF. Follow-up data were obtained from electronic medical records or phone calls to patients or family members. Follow-up duration was calculated as either the time from cardiac MRI to the occurrence of any endpoint or June 2023 (the last follow-up date).

### Statistical analysis

Statistical analyses were performed using SPSS (IBM SPSS Inc., Armonk, New York, USA) and Prism (GraphPad software Inc., San Diego, California, USA). The normality of the data was determined using the Shapiro–Wilk test. Data are expressed as the means with standard deviations or medians with interquartile ranges (IQRs) for continuous variables and frequencies for categorical variables. Comparative analyses among variables stratified by tertiles of thoracic SMI were carried out according to the type of variable using one-way analysis of variance, followed by the Bonferroni’s post hoc test or its nonparametric equivalents (Kruskal–Wallis test), chi–square test or Fisher’s exact test, as appropriate. Between-group differences in thoracic SMI in patients with and without primary outcomes were assessed using unpaired t–tests or Mann–Whitney U–tests. NT-proBNP was log-transformed and analyzed in a continuous fashion. Bivariate correlations related to thoracic SMI or NT-proBNP were obtained using the Pearson method. The relationship of NT-proBNP with thoracic SMI was assessed using linear regression analysis, and the standardized betas (β) were provided. Long-term adverse outcomes were assessed using Kaplan–Meier survival analysis and compared among different tertiles of thoracic SMI using the log-rank test. Associations of thoracic SMI and its components with adverse outcomes were determined using a multivariable Cox proportional hazards model after adjusting for potential confounders. Differences with a two-tailed P value < 0.05 were considered indicative of statistical significance.

## Results

### Baseline characteristics

Detailed baseline characteristics of the study population stratified by thoracic SMI are displayed in Table 1. There was no difference in age, sex, baseline blood pressure, heart rate, or history of smoking and drinking among the three groups (all P > 0.05), except body mass index (BMI), which was higher in patients in the third tertile (P = 0.006). HF duration, New York Heart Association functional class, etiology of HF, and comorbidity burden with the use of relevant medications were similar across groups (all P > 0.05).

**Table 1:** Baseline characteristics of the study population according to tertiles of thoracic SMI

Variables	First tertile (n = 81)	Second tertile (n = 81)	Third tertile (n = 81)
Age, yrs	57.8 ± 11.4	57.4 ± 10.9	55.7 ± 11.9
Male, n (%)	53 (65.4)	56 (69.1)	62 (76.5)
BMI, kg/m^2^	23.7 ± 3.8	24.7 ± 3.8	25.5 ± 3.3*
SBP, mmHg	120.4 ± 20.9	118.0 ± 20.5	122.0 ± 20.9
DBP, mmHg	78.2 ± 15.9	77.9 ± 14.5	80.5 ± 14.8
HR, beats/min	85.9 ± 14.2	87.4 ± 18.3	85.6 ± 19.1
Smoking, n (%)	39 (48.1)	38 (46.9)	45 (55.6)
Drinking, n (%)	27 (33.3)	32 (39.5)	31 (38.3)
HF duration, n (%)			
≤ 1 yr	46 (56.8)	40 (49.4)	49 (60.5)
> 1 and ≤ 5 yrs	22 (27.2)	21 (25.9)	25 (30.9)
> 5 yrs	13 (16.0)	20 (24.7)	7 (8.6)
NYHA functional class III– IV, n (%)	68 (80.2)	60 (74.1)	58 (71.6)
Etiology of HF			
Ischemia	25 (30.9)	29 (35.8)	24 (29.6)
Cardiomyopathy	36 (44.4)	32 (39.5)	44 (54.3)
Other	20 (24.7)	20 (24.7)	13 (16.1)
Medical history, n (%)			
HT	38 (46.9)	36 (44.4)	43 (53.1)
AF	16 (19.8)	18 (22.2)	11 (13.6)
Dyslipidemia	28 (34.6)	34 (42.0)	33 (40.7)
LBBB	5 (6.3)	9 (11.1)	5 (6.3)
COPD	11 (13.6)	4 (4.9)	7 (8.6)
SAS	1 (1.2)	4 (4.9)	4 (4.9)
Laboratory measurements			
NT‑proBNP, pg/mL	3281 (1645, 9909)	2628 (976, 4958) &	1709 (669, 3967) &#
eGFR, mL/min/1.73m^2^	67.5 ± 28.3	74.6 ± 21.5	77.1 ± 23.2*
FBG, mmol/L	8.2 (6.7, 10.5)	7.9 (6.5, 10.1)	7.3 (6.2, 9.2)
HbA1C, %	7.4 (6.6, 8.4)	7.0 (6.2, 8.0)	6.8 (6.3, 7.9)
Albumin, g/L	38.9 ± 5.3	41.6 ± 4.1*	41.7 ± 4.2*
TG, mmol/L	1.4 (1.0, 2.0)	1.6 (1.1, 2.5)	1.6 (1.1, 2.3)
TC, mmol/L	4.1 (3.5, 4.7)	3.9 (3.1, 4.6)	4.1 (3.3, 4.8)
HDL‑C, mmol/L	1.1 ± 0.4	1.0 ± 0.3	1.0 ± 0.3
LDL‑C, mmol/L	2.4 (1.9, 2.9)	2.2 (1.6, 2.9)	2.5 (1.8, 2.9)
Hemoglobin, g/L	132.7 ± 26.3	135.8 ± 22.7*	145.3 ± 22.3*†
Cardiovascular medications, n (%)			
Beta‑blocker	60 (74.1)	65 (80.2)	65 (80.2)
ACEI/ARB	57 (70.4)	63 (77.8)	64 (79.0)
SGLT-2i	27 (33.3)	26 (32.1)	30 (37.0)
Diuretics	68 (84.0)	69 (85.2)	67 (82.7)
ARNI	31 (38.3)	27 (33.3)	34 (41.9)
CCB	11 (13.6)	7 (8.6)	14 (17.3)
Anti‑thrombotic agents	43 (53.1)	45 (55.6)	49 (60.5)
Statins	38 (46.9)	39 (48.1)	41 (50.6)
Digoxin	20 (24.7)	20 (24.7)	8 (9.9) §λ

Data are presented as mean ± SD, media (Q1, Q3) or number (percentage)

One-way analysis of variance test: * P-value < 0.017 versus patients in the first tertile. † P-value < 0.017 versus patients in the second tertile. Kruskal-Wallis test: & P-value < 0.05 versus patients in the first tertile. # P-value < 0.05 versus patients in the second tertile. Chi-square test (Fisher’s exact test): § P-value < 0.05 versus patients in the first tertile. λ P-value < 0.05 versus patients in the second tertile

Abbreviations: SMI, skeletal muscle index; BMI, body mass index; SBP, systolic blood pressure; DBP, diastolic blood pressure; HR, heart rate; HF, heart failure; NYHA, New York Heart Association; HT, hypertension; AF, atrial fibrillation; COPD, chronic obstructive pulmonary disease; LBBB, complete left bundle branch block; SAS, sleep apnea syndrome; NT-proBNP, amino-terminal pro-B-type natriuretic peptide; eGFR, estimated glomerular filtration rate; FBG, fasting blood glucose; HbA1C, glycated hemoglobin; TG, triglycerides; TC, cholesterol; HDL-C, high-density lipoprotein cholesterol content; LDL‑C, low-density lipoprotein cholesterol content. ACEI, angiotensin converting enzyme inhibitor; ARB, angiotensin receptor blocker; SGLT-2i, sodium-glucose cotransporter-2 inhibitors; ARNI, angiotensin receptor-neprilysin inhibitor; CCB, calcium-channel blocker

Patients in the first tertile had the highest level of NT‑proBNP (median, 3281 [Q1-Q3, 1645–9909] pg/mL vs. 2628 [976, 4958] pg/mL vs. 1709 [669, 3967] pg/mL; P < 0.001) but the lowest estimated glomerular filtration rate (eGFR), albumin and hemoglobin levels (all P < 0.05). Moreover, compared with patients in the second and third tertiles, those in the first tertile tended to have the highest levels of fasting blood glucose (P = 0.07) and glycated hemoglobin (P = 0.09).

### Differences in cardiac MRI findings stratified by thoracic SMI

**Fig. 2:**
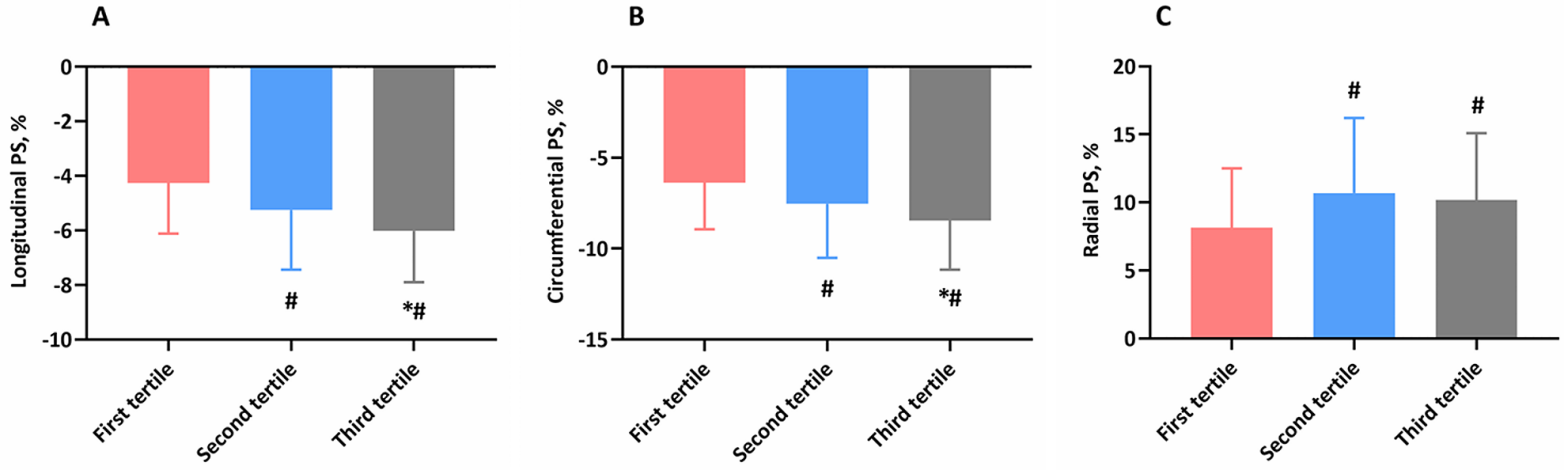
Differences of magnitude of global left ventricular systolic PS across the groups. Abbreviations: PS, peak strain. *, P value < 0.017 versus patients in the second tertile. #, P value < 0.017 versus patients in the first tertile

Although LV volumetric indices and LVEF were similar among the thoracic SMI tertiles, lower thoracic SMI occurred with a deteriorated magnitude of LV global PS in longitudinal (-4.2 ± 1.8% vs. -5.2 ± 2.1% vs. -6.0 ± 1.9%; P < 0.001), circumferential (-6.3 ± 2.5% vs. -7.5 ± 2.9% vs. -8.4 ± 2.7%; P < 0.001), and radial (8.1 ± 4.3% vs. 10.7 ± 5.5% ± 10.2 ± 4.9%; P = 0.003) components (Fig. 2). To explore the association between thoracic SMI tertiles and LV contractile function more carefully, we made subgroup analysis excluding patients with ischemic etiology. As expected, greatest deterioration of the magnitude LV global PS in longitudinal (-4.5 ± 2.1% vs. -5.6 ± 2.6% vs. -5.9 ± 1.8%; P = 0.002), circumferential (-6.5 ± 2.7% vs. -7.8 ± 3.2% vs. -7.9 ± 2.7%; P = 0.014), and radial (8.4 ± 5.3% vs. 10.7 ± 4.8% vs. 9.8 ± 5.4%; P = 0.047) components was observed in patients in the first tertile.

Interestingly, concomitant with the thoracic SMI decrease, there was an increase in LVM (153.9 ± 37.2 g vs. 139.9 ± 31.5 g vs. 126.3 ± 23.1 g; P < 0.001). The significant difference remained robust even when taking body size into account (92.3 ± 18.0 g/m^2^ vs. 81.8 ± 18.3 g/m^2^ vs. 72.8 ± 14.7 g/m^2^; P < 0.001) (Table 2). Further Pearson analysis showed a negative correlation between thoracic SMI and LVM index (r = -0.40; P < 0.001). Moreover, despite a nonsignificant distribution of myocardial scars, a higher median of scar extent in patients with LGE was observed with decreasing thoracic SMI grade (median, 27.8 [IQR, 19.3–39.7] % vs. 23.3 [16.1–31.6] % vs. 20.6 [13.6–30.8] %; P = 0.03). Mitral regurgitation occurred more frequently in patients with the lowest grade of thoracic SMI (64.2% vs. 48.1% vs. 45.7%; P = 0.04).

**Table 2:** MRI findings of the study population according to tertiles of thoracic SMI

Variables	First tertile (n = 81)	Second tertile (n = 81)	Third tertile (n = 81)
LVEDV, mL	262.8 (185.8, 339.3)	256.2 (176.4, 316.5)	260.8 (197.9, 308.3)
LVEDV index, mL/m^2^	152.8 (125.7, 195.5)	148.4 (109.7, 185.7)	147.0 (123.0, 173.8)
LVESV, mL	199.7 (129.3, 285.5)	190.9 (118.7, 261.6)	193.8 (143.4, 239.1)
LVESV index, mL/m^2^	121.7 (89.4, 163.8)	108.9 (71.2, 147.6)	111.3 (83.2, 135.4)
LVSV, mL	55.4 (39.4, 72.3)	56.3 (45.9, 77.5)	63.1 (47.7, 77.0)
LVSV index, mL/m^2^	32.5 (24.6, 44.5)	35.6 (25.9, 45.4)	36.1 (28.4, 45.0)
LVEF, %	22.8 ± 8.3	26.9 ± 8.8	26.7 ± 9.1
LVM, g	153.9 ± 37.2	139.9 ± 31.5*	126.3 ± 23.1*†
LVM index, g/m^2^	92.3 ± 18.0	81.8 ± 18.3*	72.8 ± 14.7*†
MR, n (%)	52 (64.2)	39 (48.1) §	37 (45.7) §
LGE present, n (%)	55 (67.9)	54 (66.7)	60 (74.1)
LGE size, % LVM	27.8 (19.3, 39.7)	23.3 (16.1, 31.6) &	20.6 (13.6, 30.8) &#

Data are presented as mean ± SD, media (Q1, Q3) or number (percentage)

One-way analysis of variance test: * P-value < 0.017 versus patients in the first tertile. † P-value < 0.017 versus patients in the second tertile. Kruskal-Wallis test: & P-value < 0.05 versus patients in the first tertile. # P-value < 0.05 versus patients in the second tertile. Chi-square test (Fisher’s exact test): § P-value < 0.05 versus patients in the first tertile

Abbreviations: SMI, skeletal muscle index; LVEDV, left ventricular end-diastolic volume; LVESV, left ventricular end-systolic volume; LVSV, left ventricular stroke volume; LVEF, left ventricular ejection fraction; LVM, left ventricular mass; MR, mitral regurgitation; LGE, late gadolinium enhancement

### Associations of NT-proBNP with thoracic SMI and BMI

**Fig. 3:**
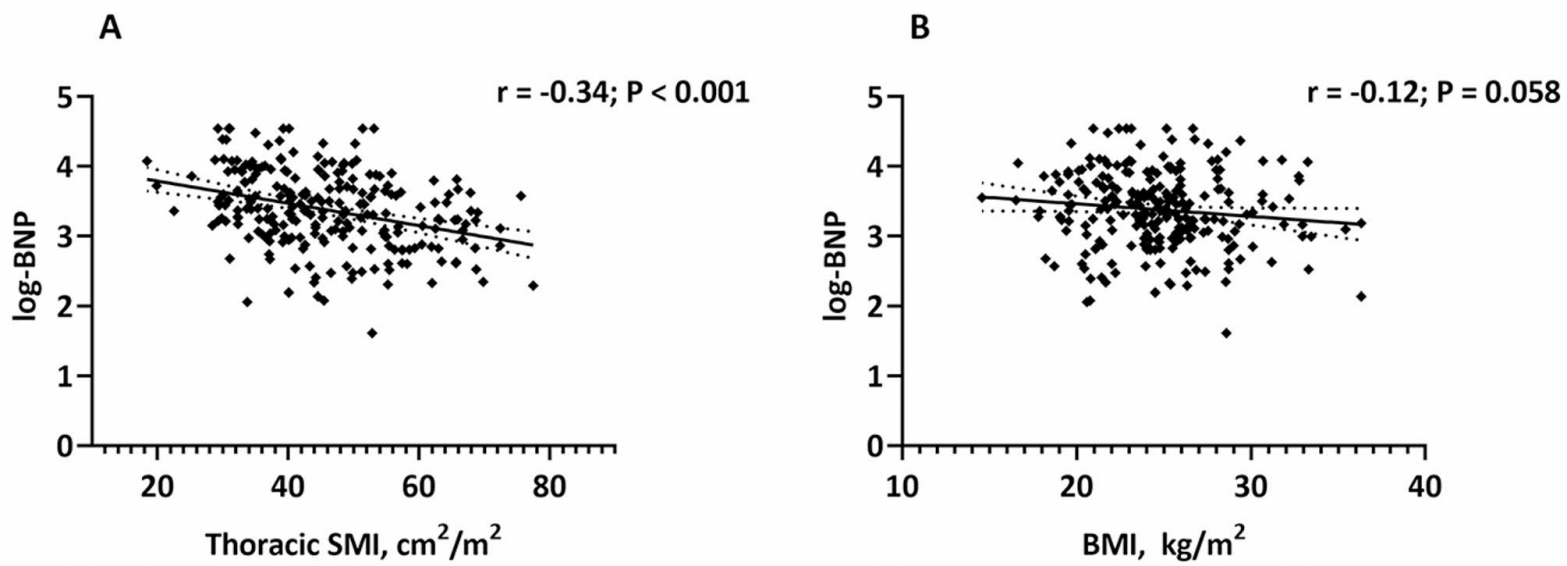
Bivariate correlations between NT-proBNP and thoracic SMI or BMI. NT-proBNP is log-transformed before being included in the Pearson’s analysis. Abbreviations: NT-proBNP, amino-terminal pro-B-type natriuretic peptide; SMI, skeletal muscle index; BMI, body mass index

As demonstrated in Fig. 3, we found an inverse correlation between NT-proBNP and thoracic SMI (r = -0.34; P < 0.001), whereas only a weak trend was obtained between NT-proBNP and BMI (r = -0.12; P = 0.06). These relationships were assessed with linear regression. In the unadjusted univariate analysis, thoracic SMI (β = -0.34; P < 0.001), rather than BMI (β = -0.12; P = 0.06), was associated with NT-proBNP. In a model that adjusted for age, sex, NYHA functional class, the eGFR, LVEF and LVM index, the association between thoracic SMI and NT-proBNP remained unchanged (β = -0.25; P < 0.001) but not the association with BMI (β = -0.04; P = 0.55) (Table 3).

**Table 3:** Linear regression analysis for factors associated with NT-proBNP

Variables	Univariable coefficient B (one unit increase for continuous variables/yes or no for categorical variables)	Univariable standardized coefficient β (compare the effect estimates)	Multivariable coefficient B (one unit increase for continuous variables/yes or no for categorical variables)	Multivariable standardized coefficient β (adjusted R^2^ = 0.31) (compare the effect estimates)
Age	0.01	0.20†	0.004	0.08†
Male sex	-0.08	-0.07		
BMI	-0.02	-0.12		
NYHA functional class ≥ III	0.46	0.27†	0.456	0.25†
eGFR	-0.01	-0.39†	-0.007	-0.32†
LVEF	-0.01	-0.13†	-0.006	-0.11†
LVM index	0.01	0.31†	0.006	0.20†
Thoracic SMI	-0.02	-0.34†	-0.007	-0.25†

Abbreviations: NT-proBNP, amino-terminal pro-B-type natriuretic peptide; BMI, body mass index; NYHA, New York Heart Association; eGFR, estimated glomerular filtration rate; LVEF, left ventricular ejection fraction; LVM, left ventricular mass; SMI, skeletal muscle index

† indicates P-value < 0.05. NT-proBNP is log-transformed before being included in the regression analysis

### Associations of thoracic SMI and its components with clinical outcomes

**Fig. 4:**
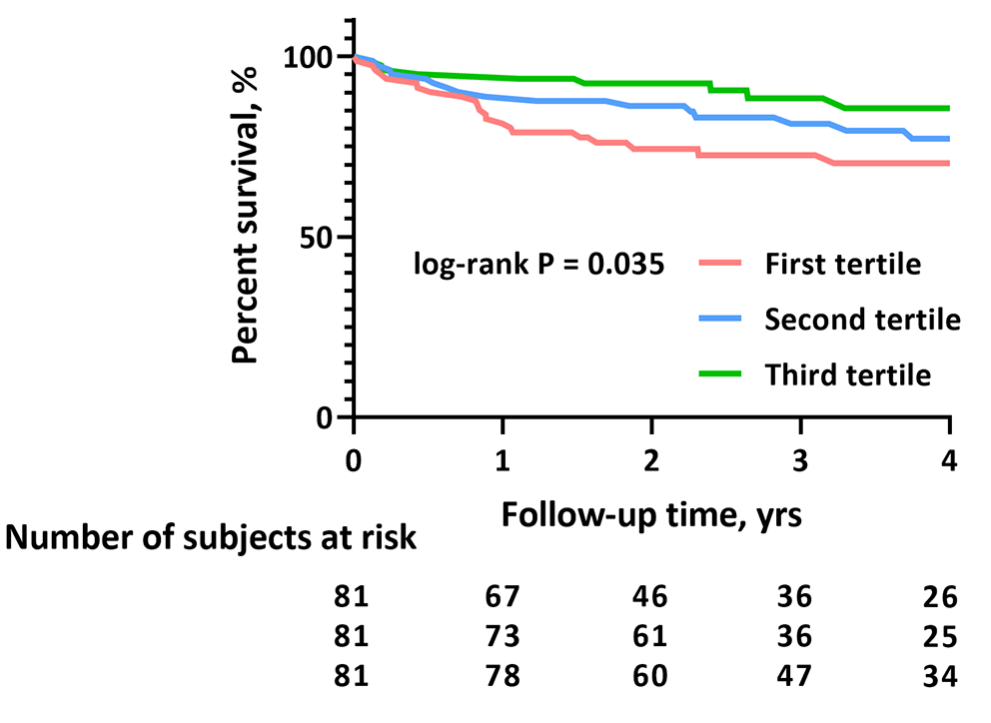
Survival curves of the study cohort according to tertiles of thoracic SMI. Abbreviations: SMI, skeletal muscle index

During a median follow-up of 33.6 months (Q1-Q3, 20.4–52.8 months), a total of 48 patients (19.8%) met one confirmed composite endpoint event. The primary composite events occurred more frequently in patients in the lowest grade of thoracic SMI (27.2% vs. 21.0% vs. 11.1%; P = 0.035). By Kaplan–Meier survival analysis, patients in the lowest tertile, indicating the lowest size of thoracic skeletal muscle, were more likely to experience the primary outcome than those in the middle and highest tertiles during follow-up (log-rank P = 0.035; Fig. 4).

Patients with adverse outcomes had reduced thoracic SMI (median, 40.1 [IQR, 34.3–47.9] cm^2^/m^2^ vs. 45.3 [37.3–55.0] cm^2^/m^2^; P = 0.017), pectoralis major index (11.0 [8.8–14.3] cm^2^/m^2^ vs. 10.2 [8.1–12.0] cm^2^/m^2^; P = 0.019), and periscapular index (20.7 [15.0-27.3] cm^2^/m^2^ vs. 17.4 [13.7–22.6] cm^2^/m^2^; P = 0.029) compared with those without adverse outcomes. Nevertheless, BMI in patients with adverse outcomes was comparable to that in patients without (24.8 ± 3.9 kg/m^2^ vs. 24.6 ± 3.7 kg/m^2^; P = 0.76). Our multivariable Cox proportional hazards model showed that a higher thoracic SMI (HR: 0.96; 95% CI: 0.92–0.99; P = 0.01), pectoralis major index (HR: 0.85; 95% CI: 0.75–0.96; P < 0.01), and periscapular index (HR: 0.94; 95% CI: 0.90–0.99; P = 0.02) were each independently associated with a lower risk of the primary outcome in diabetes patients with HFrEF (Table 4). However, our analysis did not yield any associations between BMI and the primary outcome in the same condition.

**Table 4:** Associations of thoracic SMI and its components with primary outcomes in the study cohort

Variables	Patients without primary outcomes (n = 48)	Patients with primary outcomes (n = 195)	Unadjusted HR (95% CI)	Adjusted HR^a^ (95% CI)
Thoracic SMI, cm^2^/m^2^	45.3 (37.3, 55.0)	40.1 (34.3, 47.9) ^b^	0.96 (0.94, 0.99) [0.01]	0.96 (0.92, 0.99) [0.01]
Pectoralis major index, cm^2^/m^2^	11.0 (8.8, 14.3)	10.2 (8.1, 12.0) ^b^	0.87 (0.80, 0.95) [< 0.01]	0.85 (0.75, 0.96) [< 0.01]
pectoralis minor index, cm^2^/m^2^	2.2 (1.6, 3.0)	2.2 (1.6, 2.7)	0.83 (0.61, 1.14) [0.25]	0.86 (0.60, 1.24) [0.42]
serratus anterior index, cm^2^/m^2^	4.0 (3.3, 4.8)	4.0 (3.2, 4.9)	1.09 (0.89, 1.33) [0.40]	1.10 (0.90, 1.33) [0.37]
Periscapular index, cm^2^/m^2^	20.7 (15.0, 27.3)	17.4 (13.7, 22.6) ^b^	0.95 (0.91, 0.99) [0.03]	0.94 (0.90, 0.99) [0.02]
Paraspinal index, cm^2^/m^2^	4.4 ± 1.3	4.3 ± 1.2	0.89 (0.70, 1.13) [0.34]	0.85 (0.65, 1.12) [0.25]
Trapezius index, cm^2^/m^2^	2.4 (2.0, 2.9)	2.3 (1.9, 2.8)	0.81 (0.53, 1.23) [0.33]	0.83 (0.51, 1.34) [0.44]

Data are presented as media (Q1, Q3) or mean ± SD. Data in brackets are P-values

Abbreviations: SMI, skeletal muscle index; HR, hazards ratio; CI, confidence interval; BMI, body mass index; SBP, systolic blood pressure; HF, heart failure; NT-proBNP, amino-terminal pro-B-type natriuretic peptide; eGFR, estimated glomerular filtration rate; SGLT-2i, sodium-glucose cotransporter-2 inhibitors; LVEF, left ventricular ejection fraction; LVM, left ventricular mass

a. Based on a multivariable Cox model adjusted for age, sex, BMI, SBP, etiology of HF (ischemic or not), NYHA functional class, NT-proBNP, eGFR, use of SGLT-2i, LVEF and LVM index

b. Mann-Whitney test: P-value < 0.05

## Discussion

The present study emphasized the necessity of thoracic SMI evaluation and provided a convenient surrogate for sarcopenia with prognostic information in the routine care of diabetes patients with HFrEF. The principal findings were as follows: (1) in diabetes patients with HFrEF, a reduction in thoracic SMI was more likely to be associated with a deterioration in LV contractility, together with an increase in LVM and a heavier burden of myocardial fibrosis, leading to “nonfunctional” cardiac hypertrophy; (2) thoracic SMI, rather than BMI, was independently associated with the level of NT-proBNP; and (3) a lower thoracic SMI indicated a higher risk of adverse clinical outcomes, regardless of cardiac functional/structural measurements. Thoracic SMI and its components assessed by routine cardiac MRI could be used as a new predictor of outcomes in diabetes patients with HFrEF.

### Role of sarcopenia in LV remodeling

The role of sarcopenia in the context of HF has recently received much attention. This change in body composition is considered a key determinant of many symptoms related to HF syndrome and is associated with poor outcomes, suggesting the detrimental effect of sarcopenia on LV remodeling in patients with chronic HF [4, 5, 11–14]. However, these studies fail to further reveal the alterations of LV structure and function in a given HF phenotype, and the possible cardiac remodeling in the condition of skeletal muscle wasting is incompletely understood. In contrast, with respect to diabetes patients with HFrEF, our data showed that those with reduced axial thoracic muscle mass were more likely to display deteriorated LV contractility despite similar LVEF. Indeed, it is believed that both sarcopenia and HF interact with each other, with the onset of one disease promoting disease progression in the other. Our findings may partially be explained by the mechanism by which hypoperfusion accompanied by LV dysfunction in HFrEF could induce skeletal myopathy, which subsequently yields increased activation of the sympathetic nervous system as well as the renin–angiotensin–aldosterone system and consequently leads to endothelial dysfunction, vasoconstriction and myocardial injury [3, 15–17].

Interestingly, we found that patients in the lowest tertile of thoracic SMI exhibited a higher LVM with a higher burden of myocardial fibrosis. In terms of this point, only the study by Beyer et al. reported similar findings in UK adults, which indicated an inverse correlation between the severity of sarcopenia and cardiac mass [18]. The role of sarcopenia in the development of cardiac remodeling in HFrEF is not fully understood. Apart from the abovementioned validated mechanisms, it has also been observed that sarcopenia and HFrEF share similar proinflammatory cytokines and molecular pathways in the occurrence and persistence of myocardial inflammation, which together are sufficient to cause cardiomyocyte death and secondary myocardial fibrosis [19–22]. Thus, in view of the characteristics of “nonfunctional” cardiac hypertrophy accompanied by cardiac dysfunction and remarkable myocardial fibrosis, whether a sarcopenic heart exists needs further study for clarification.

### Prognostic ability of axial thoracic SMI

The “obesity paradox” is a well-known phenomenon in established HF that describes a survival benefit in obese patients. However, for diabetes patients comorbid with HFrEF, data from multicenter studies suggested that obesity may confer no advantage at all to this patient group [23–25]. To date, there is no consensus on the reason why the obesity paradox is absent in diabetes patients with HFrEF. A previous report observed significantly lower sympathetic activation in obese patients than in their nonobese counterparts, but whether the positive impact of attenuated sympathetic activation in obese patients with HF is blunted by the presence of DM remains unknown [26]. In the present study, axial thoracic SMI, but not BMI, demonstrated an independent relationship with NT-proBNP, and only a reduction in axial thoracic SMI indicated an increased risk of adverse outcomes. Based on these findings, we speculate that a superimposed and synergistic effect exerted by the coexistence of DM and sarcopenia may promote the process of adverse LV remodeling, leading to poor outcomes in HFrEF. In fact, the simple BMI measurement is unable to reflect the changes in body composition and thereby cannot separate lean mass from fat mass. Moreover, skeletal muscle wasting often occurs prior to adipose loss [4, 19]. In this view, the present study may help elucidate why BMI is not independently associated with survival in diabetes patients with HFrEF. Therefore, our data highlight the importance and sensitivity of skeletal muscle mass changes in relation to prognosis in this condition.

Consistent with published literature, the cross-sectional size of the pectoralis major in this study was demonstrated to be an independent predictor of clinical outcomes [4, 13, 27]. Moreover, our study additionally evaluated and identified the periscapular muscle groups, which were found to be the other major muscle groups with prognostic value. The possible explanation for this observation is that the wasting of these skeletal muscle groups is more reflective of physical performance, such as the maintenance of upright posture and cardio-pulmonary function, which itself is an indicator of the development of frailty. Further studies in larger and more diverse populations are needed to confirm our findings.

### Evaluation of axial thoracic SMI by routine cardiac MRI in clinical practice

Currently, the European Working Group on Sarcopenia in Older People 2 (EWGSOP2) recommends dual-energy X-ray absorptiometry (DEXA) as the method of choice for the evaluation of skeletal muscle quantity in clinical settings. Although this method has the advantages of being fast and low-cost, the results are susceptible to fluid status, which frequently fluctuates with HF, making it less useful in these cases [6]. Moreover, this method has been traditionally applied in assessing skeletal muscle of the upper or lower extremities, which is more subject to deconditioning [12, 28]. Our methods of quantification of thoracic skeletal muscle mass suggest that it is feasible to obtain important prognostic information related to sarcopenia from routine cardiac MRI images alone without adding additional sequences. Thus, our study may provide a “one-stop” scanning protocol not only for cardiac structure and function but also for sarcopenia assessment in diabetes patients with HFrEF.

### Study limitations

The present study had some limitations. First, we identified the prognostic ability of thoracic skeletal muscle size for diabetes patients with HFrEF based on the SMI tertile. However, the threshold for this novel predictor to define low muscle mass is currently unknown. Further studies should be conducted in HF populations to confirm the specific cutoff value of thoracic skeletal muscle size for the diagnosis of sarcopenia. Second, although our study found a novel and promising alternative for assessing reduced skeletal muscle, we did not include functional data, such as muscle strength and physical performance, which is helpful in stratification. This issue merits further investigation. Finally, we must acknowledge that due to the retrospective nature of this study, selection bias was inevitable.

In conclusion, thoracic SMI, but not BMI, is independently associated with adverse outcomes in diabetes patients with HFrEF and is a surrogate of sarcopenia that can be obtained by a readily available routine cardiac MRI protocol. Our study provided a novel prognostic predictor and highlighted the necessity of assessing thoracic skeletal muscle size in diabetes patients with HFrEF. Further studies with more sample sizes are warranted to validate our findings.

## Data Availability

The datasets used and/or analyzed during the current study are available from the corresponding author on reasonable request.

## Abbreviations

HF: heart failure
DM: diabetes mellitus
HFrEF: heart failure with reduced ejection fraction
LV: left ventricular
NT-proBNP: amino-terminal pro-B-type natriuretic peptide
LVEF: left ventricular ejection fraction
SSFP: steady-state free precession
TE: echo time
FA: flip angle
FOV: field of view
TR: repetition time
LGE: late gadolinium enhancement
SMI: skeletal muscle index
EDV: end-diastolic volume
ESV: end-systolic volume
SV: stroke volume
LVM: left ventricular mass
PS: peak strain
IQRs: interquartile ranges
BMI: body mass index
eGFR: estimated glomerular filtration rate
